# The burden of septic arthritis on the U.S. inpatient care: A national study

**DOI:** 10.1371/journal.pone.0182577

**Published:** 2017-08-15

**Authors:** Jasvinder A. Singh, Shaohua Yu

**Affiliations:** 1 Medicine Service, Birmingham VA Medical Center, Birmingham, Alabama, United States of America; 2 Department of Medicine at School of Medicine, and Division of Epidemiology at School of Public Health, University of Alabama at Birmingham (UAB), Birmingham, Alabama, United States of America; 3 Department of Orthopedic Surgery, Mayo Clinic College of Medicine, Rochester, Minnesota, United States of America; Straub Clinic and Hospital, UNITED STATES

## Abstract

**Objective:**

To assess the health care burden of septic arthritis in the U.S. and examine the associated factors.

**Methods:**

We used the U.S. Nationwide Emergency Department Sample (NEDS) data of patients hospitalized with septic arthritis as the primary diagnosis from 2009–12 to assess time-trends. Multivariable-adjusted models assessed demographics, comorbidity and hospital characteristics as potential predictors of duration of hospitalization, total hospital (inpatient and ED) charges and discharge to home.

**Results:**

In 2009, 2010 and 2012 in the U.S., respectively, there were 13,087, 13,662 and 13,714 hospitalizations with septic arthritis as the primary diagnosis. Respective average hospital stay was 7.4 vs. 7.4 vs. 7.2 days; total hospital charges were $601 vs. $674 vs. $759 million; and proportion discharged home were 43% vs. 43% vs. 44%. Almost 25% each were discharged to a skilled facility or with home health. Age >50 years, Medicaid and self-pay as primary payer, Northeast U.S. hospital location, teaching hospital status, heart failure and diabetes were associated with longer hospitalization; hyperlipidemia, hypertension or gout were associated with a shorter hospital stay. Similar associations were noted for higher hospital charges. Age >50 years, higher income, Medicare insurance, heart failure, diabetes and longer hospital stay were associated with lower odds, and Western U.S. hospital location and gout with higher odds, of discharge to home.

**Conclusions:**

We noted an increase in hospital charges from 2009–12, but no time trends in duration or outcomes of hospitalization for septic arthritis. Comorbidity associations with outcomes indicate the potential for developing interventions to improve outcomes.

## Introduction

Septic arthritis is a serious condition associated with significant morbidity and mortality [1]. It is frequently treated with intravenous antibiotics and surgical drainage of the joint, often managed initially in an inpatient setting; associated complications can lead to the need for additional surgeries and significant morbidity and disability [2]. A U.S. study examined a national sample of children with septic arthritis [3]. To our knowledge, no national estimates of health care burden of septic arthritis in adults are available in the U.S., or other countries. Thus, very little is known about health care utilization by adults with septic arthritis, which is primarily in an inpatient setting.

We conducted a PubMed search using the keywords “septic arthritis” and “hospitalization” on 11/27/16 and found 256 titles. None of the studies examined outcomes after hospitalization in adults with septic arthritis, and most were single center case series. Therefore, we do not know the inpatient utilization associated with septic arthritis and whether it has changed over time.

Medical comorbidities such as diabetes, osteoarthritis and inflammatory arthritis are risk factors for incident septic arthritis. An important question is whether their presence contributes to the inpatient resource utilization in patients who are admitted to the hospital with septic arthritis. We also do not know whether health care facility factors (type, location etc.) or patient’s socioeconomic status and demographics influence the associated inpatient utilization. Our aim was to address this knowledge gap by utilizing a national U.S. sample. The objective of our retrospective cohort study was to examine patients who were hospitalized for septic arthritis to: (1) assess whether demographics, comorbidity, and facility-level factors were associated with longer durations of hospital stay or higher hospital charges; (2) understand the factors associated with discharge to home after hospitalization; and (3) examine whether the hospitalization rates, associated charges and disposition after hospitalization had changed over time.

## Materials and methods

### Source and study population

We used the Nationwide Emergency Department Sample (NEDS) for years 2009, 2010 and 2012 for this study; 2011 data could not be analyzed due to data duplication at the time of data analysis. NEDS data are provided for public use by the Healthcare Cost and Utilization Project (HCUP), supported by the Agency for Healthcare Research and Quality (AHRQ). NEDS contains a 20-percent stratified sample of all ED visits in the U.S. [4, 5]; appropriate weights are provided to users to obtain weighted national estimates. Approximately 950 U.S. hospitals from 30 states contributed data in 2012. NEDS data originate from two databases: (1) the State Emergency Department Databases (SEDD) that capture discharge information on all ED visits that do not result in an admission to the same hospital; and (2) the State Inpatient Databases (SID) that capture discharge information on all ED visits that result in an admission to the same hospital [4, 5]. NEDS contains event-level data, and not patient-level data.

We defined our study cohort as adults (aged 18 and older) with hospitalization with septic arthritis as the primary diagnosis after an ED visit with septic arthritis. We identified septic arthritis using the International Classification of Diseases, ninth revision, Common Modification (ICD-9-CM) code of 711.0 or 711.0x, a valid approach for identifying patients with septic arthritis [6].

For each ED visit that resulted in a hospitalization, NEDS includes up to 15 ICD-9-CM diagnostic codes and 9 ICD-procedure codes (diagnoses and procedures). Diagnostic codes are listed in primary (first) or secondary positions (2–15). For this study, we included hospitalizations with septic arthritis as the primary diagnosis, and most analyses analyzed this patient population. For estimation of descriptive statistics, we also analyzed hospitalizations with septic arthritis as primary or secondary diagnosis. The Institutional Review Board at the University of Alabama at Birmingham (UAB) approved the study and all investigations were conducted in conformity with ethical principles of research.

### Outcomes of interest

We examined three outcomes of interest for hospitalizations with septic arthritis as the primary diagnosis. Outcomes were the duration of hospitalization, total hospital (ED and inpatient) charges, discharge to home after hospitalization with septic arthritis as the primary diagnosis.

### Covariates

Several demographic (including socioeconomic/health access), comorbidity and hospital characteristics were included as covariates and we adjusted for them in multivariable-adjusted analyses. Demographics included age, sex, insurance type, residence (urban vs. rural) and the annual median household income, which was estimated using the residential zip code. Comorbidities included coronary heart disease (CHD), hypertension, hyperlipidemia, renal failure, heart failure, gout, diabetes, chronic obstructive pulmonary disease (COPD) and osteoarthritis (OA), either previously known to be associated with septic arthritis and/or due to common occurrence. We included hospital characteristics such as geographical region (Northeast, Midwest, South and West), location in metropolitan or non-metropolitan area, and whether it was a teaching or a non-teaching hospital.

### Statistical analysis

We calculated the duration of hospitalization, total (ED and inpatient) and inpatient charges, charges per visit, and disposition after hospitalization for patients with hospitalizations with septic arthritis from 2009, 2010 and 2012, for hospitalizations with septic arthritis as primary diagnosis and for hospitalizations with septic arthritis as primary or secondary diagnosis. The original 2011 NEDS included duplicated records in the South and Midwest and a corrected 2011 dataset was not available in an amended form at the time of analysis. We calculated estimates of central tendency (mean, median) and variation (standard errors, inter-quartile range). All other analyses were done only for hospitalizations with septic arthritis as the primary diagnosis. Characteristics of patients admitted vs. not admitted after an ED visit were compared using chi-square or student’s t-test.

We decided *a priori* to use the 2012 NEDS data for all univariate and multivariable-adjusted analyses for duration of hospitalization, total hospital charges, and discharge to home after hospitalization. We made this decision since 2012 NEDS data were the most recent data available that had enough events for us to perform these analyses. We examined admissions for procedures used for diagnosis and/or treatment of septic arthritis: arthrocentesis, 81.91; and arthrotomy 80.xx. We used linear regression analyses to examine the duration of hospitalization and total hospital charges for septic arthritis as the primary diagnosis. We used logistic regression for assessing discharge to home after hospitalization for septic arthritis as the primary diagnosis. Multivariable-adjusted linear (duration of hospitalization, ED charges), or logistic regression (discharge to home) included demographic, comorbidity and hospital characteristics, and was performed using SAS version 9.3 (SAS corporation, Cary, NC, USA). We calculated beta-estimates or odds ratios (OR) with 95% confidence intervals (CI). Sensitivity analyses were done using the logarithm of duration of hospital stay or total charges, which were normally distributed, to account for the slight skewness of duration or charges data.

## Results

### Time trends in charges, duration of stay and disposition after hospitalization

There were 13,087, 13,662 and 13,714 hospitalizations with septic arthritis as the primary diagnosis in the U.S. in 2009, 2010 and 2012, respectively (Table 1). The average duration of hospital stay for septic arthritis did not change significantly over years, 7.4 vs. 7.4 vs. 7.2 days, although median length of stay seemed to decrease slightly, 5.1 vs. 4.8 vs. 4.6 days, respectively. We noted an increase in total charges (inpatient and ED) over time, $601 vs. $674 vs. $759 million, respectively (Table 1). The inpatient portion of the charges for septic arthritis was $538 million in 2009 that increased to $667 million in 2012 in the U.S. (Table 1). The total ED and inpatient charges for all admissions with septic arthritis as primary or secondary diagnosis were $2.0, $2.3 and $2.8 billion in 2009, 2010 and 2012, respectively.

**Table 1:** Descriptive statistics for utilization and charges for hospitalizations with septic arthritis.

	2009	2010	2012
**Septic arthritis as the primary diagnosis**			
Number of Inpatient admissions	13,087	13,632	13,714
Length of hospital stay for admitted patients, in days			
Mean (SE)	7.40 (0.16)	7.38 (0.17)	7.16 (0.18)
Median (IQR)	5.05 (2.91, 8.48)	4.79 (2.88, 8.16)	4.64 (2.80, 7.90)
Total charges per visit for admitted patients, US$)			
Mean (SE)	$45,945 (1,505)	$49,449 (1,567)	$55,354 (1,873)
Median (IQR)	$30,359 (18,105, 55,335)	$33,529 (19,284, 59,102)	$37,563 (21,357, 66,104)
Total inpatient charges ($)	$538,137,440	$609,691,200	$667,295,812
Total ED and inpatient charges ($)	$601,282,215	$674,088,768	$759,124,756
**Septic arthritis as primary or secondary diagnosis**			
Number of Inpatient admissions	31,668	33,852	36,539
Duration of hospital stay, in days	9.72 (0.17)	9.58 (0.18)	9.27 (0.18)
Total inpatient charges ($)	$1,831,170,432	$2,076,413,976	$2,523,164,106
Total ED and inpatient charges ($)	$2,031,248,856	$2,322,077,940	$2,851,138,170

SE, standard error

IQR, Inter-quartile range

ED, Emergency Department

^$^, U.S. dollar

Patients with a primary diagnosis of septic arthritis who were admitted from the ED, differed from those not admitted from ED in that they were older, had higher income, and were more likely to be female, live in a metropolitan area or Southern U.S., have Medicare as the payer or receive care at Metropolitan teaching hospital. They were also 2–5 times more likely to have each medical comorbidity compared to patients not admitted (Table 2). Of those admitted with septic arthritis as the primary diagnosis in 2012, 78% underwent arthrocentesis and/or arthrotomy.

**Table 2:** Characteristics of patients with septic arthritis ED visits who were hospitalized compared to those not hospitalized.

	2012 NEDS (all)	2012 NEDS, ED visits Not admitted N (%)*	2012 NEDS, ED visits who were admitted N (%)*	P-value, admitted vs. not admitted
**ED visits**	16,382 (100)	2,668 (16.29)	13,714 (83.71)	
Age, in years, Mean (SE)	56.28 (0.40)	51.99 (0.95)	57.11 (0.42)	<0.0001
Female Sex	5891 (35.96)	797 (29.87)	5094 (37.15)	0.003
Patient location (residence)				<0.0001
Micropolitan/not metro	2932 (18.07)	979 (36.99)	1953 (14.38)	
Metropolitan (large or small)	13290 (81.93)	1668 (63.01)	11622 (85.62)	
Median house hold income				0.046
1st quartile (< $38,999)	4752 (29.83)	842 (32.4)	3909 (29.33)	
2nd quartile ($39,000 to $47,999)	4141 (26.00)	779 (29.97)	3362 (25.22)	
3rd quartile ($48,000 to $62,999)	3851 (24.18)	536 (20.60)	3315 (24.87)	
4th quartile ($63,000 or more)	3184 (19.99)	442 (17.02)	2741 (20.57)	
Primary payer				<0.0001
Medicare	6778 (41.46)	887 (33.31)	5890 (43.04)	
Medicaid	2407 (14.73)	379 (14.23)	2028 (14.82)	
Private insurance	4071 (24.90)	722 (27.12)	3349 (24.47)	
Self-pay/No charge	2110 (12.91)	521 (19.57)	1589 (11.61)	
Other	982 (6.00)	154 (5.77)	828 (6.05)	
Hospital Region				0.089
Northeast	3371 (20.58)	532 (19.97)	2838 (20.69)	
Midwest	3680 (22.46)	674 (25.27)	3006 (21.92)	
South	5565 (33.97)	767 (28.74)	4798 (34.99)	
West	3766 (22.99)	694 (26.02)	3072 (22.40)	
Teaching status of hospital				<0.0001
Metropolitan non -teaching or non-metro	8358 (51.02)	1789 (67.07)	6568 (47.90)	
Metropolitan teaching	8024 (48.98)	879 (32.93)	7145 (52.10)	
Comorbidities				
CHD	2024 (12.35)	125 (4.67)	1899 (13.85)	<0.0001
Hypertension	7794 (47.58)	670 (25.09)	7124 (51.95)	<0.0001
Hyperlipidemia	3526 (21.53)	232 (8.71)	3294 (24.02)	<0.0001
Renal failure	2070 (12.63)	72 (2.70)	1998 (14.57)	<0.0001
CHF	1351 (8.25)	49 (1.83)	1302 (9.50)	<0.0001
Gout	1543 (9.42)	73 (2.74)	1470 (10.72)	<0.0001
Diabetes	3866 (23.60)	361 (13.54)	3505 (25.56)	<0.0001
COPD	1254 (7.65)	77 (2.87)	1177 (8.59)	<0.0001
Osteoarthritis	2029 (12.38)	68 (2.54)	1961 (14.30)	<0.0001

CHD, coronary heart disease; CHF, Congestive Heart failure; COPD, chronic obstructive pulmonary disease

*P-value **comparing not admitted vs. admitted; all comparisons were significant except hospital region**

N (%)*, unless specified otherwise

Almost half of the patients admitted with septic arthritis as the primary diagnosis were discharged home, and a quarter each were discharged to skilled nursing facility or with home health care (Table 3); results were similar for septic arthritis as primary or secondary diagnosis.

**Table 3:** Disposition of patients after hospital admission for septic arthritis.

	2009 NEDS	2010 NEDS	2012 NEDS
**Hospitalization disposition for septic arthritis as primary diagnosis**, n (%)			
Discharged home	5,619 (43.01)	5,839 (43.00)	6,046 (44.12)
Skilled nursing facility, intermediate Care facility, and another type of facility	3,334 (25.52)	3,538 (26.06)	3,550 (24.45)
Transferred to short term hospital	492.5 (3.77)	480 (3.54)	543 (3.96)
Home health care	3,198 (24.48)	3,346 (24.65)	3,329 (24.30)
Against medical advice	295 (2.26)	263 (1.94)	341 (2.49)
Died	122 (0.93)	95 (0.71)	94 (0.69)
Hospitalization disposition with Septic arthritis as primary or secondary diagnosis*, n (%)			
Discharged home	10,902 (34.48)	11,260 (33.40)	12,654 (34.67)
Skilled nursing facility, intermediate Care facility, and another type of facility	10,478 (33.14)	11,500 (34.11)	11,704 (32.06)
Transferred to short term hospital	1,372 (4.34)	1,537 (4.56)	1,749 (4.79)
Home health care	7,603 (24.05)	8,031 (23.82)	8,776 (24.04)
Against medical advice	501 (1.59)	533 (1.58)	799 (2.19)
Died	741 (2.34)	830 (2.46)	817 (2.24)

SE, standard error

*Septic arthritis listed either as primary or secondary diagnosis

### Predictors of duration of hospital stay and total charges for septic arthritis

In multivariable-adjusted analyses, age >50 years, Medicaid or self-pay as primary payer, hospital location in the Northeast region, metropolitan teaching hospital status, heart failure and diabetes were associated with a longer hospital stay (Table 4). Hyperlipidemia, hypertension or gout were associated with a shorter hospital stay (Table 4).

**Table 4:** Predictors of the duration of hospital stay among patients with septic arthritis hospitalized with septic arthritis after presenting to the ER.

	Univariate		Multivariable-adjusted	
	Beta-estimate (95% CI)	P-value	Beta-estimate (95% CI)	P-value
Age				
<50	Ref		Ref	
50- <65	**1.16 (0.37, 1.96)**	**0.004**	**1.81 (1.02, 2.61)**	**<0.0001**
65- <80	0.22 (-0.66, 1.09)	0.63	**1.81 (0.31, 3.31)**	**0.02**
≥80	-0.06 (-1.00, 0.88)	0.90	**1.58 (0.12, 3.03)**	**0.03**
Gender				
Female	Ref		Ref	
Male	0.08 (-0.46, 0.62)	0.77	0.12 (-0.40, 0.65)	0.64
Median household income				
1st quartile (< $38,999)	Ref		Ref	
2nd quartile ($39,000 to $47,999)	0.09 (-0.83, 1.02)	0.84	0.47 (-0.44, 1.38)	0.31
3rd quartile ($48,000 to $62,999)	0.27 (-0.65, 1.20)	0.56	0.51 (-0.42, 1.44)	0.28
4th quartile ($63,000 or more)	**-0.87 (-1.65, -0.08)**	**0.03**	-0.77 (-1.62, 0.09)	0.08
Primary payer				
Medicare	Ref		Ref	
Medicaid	**2.31 (1.21, 3.42)**	**<0.0001**	**2.57 (1.30, 3.85)**	**<0.0001**
Private insurance	**-0.98 (-1.45, -0.52)**	**<0.0001**	-0.45 (-1.25, 0.35)	0.27
Self-pay/No charge	1.37 (-0.05, 2.79)	0.06	**1.91 (0.34, 3.49)**	**0.02**
Other	1.82 (-0.82, 4.45)	0.18	2.14 (-0.88, 5.15)	0.16
Patient location (residence)				
Micropolitan/not metro	Ref		Ref	
Metro (large or small)	**1.36 (0.61, 2.10)**	**0.0004**	0.58 (-0.22, 1.38)	0.15
Hospital Region				
Northeast	Ref		Ref	
Midwest	**-2.07 (-3.05, -1.10)**	**<0.0001**	**-2.12 (-3.09, -1.14)**	**<0.0001**
South	**-1.24 (-2.14, -0.34)**	**0.007**	**-1.44 (-2.36, -0.53)**	**0.002**
West	**-1.39 (-2.67, -0.10)**	**0.03**	**-1.37 (-2.65, -0.09)**	**0.04**
Teaching status of hospital				
Metropolitan non -teaching or non-metro	Ref		Ref	
Metropolitan teaching	**1.69 (0.96, 2.42)**	**<0.0001**	**1.33 (0.56, 2.10)**	**0.0008**
Comorbidities				
CHD (ref: no)	0.40 (-0.66, 1.46)	0.46	0.47 (-0.71, 1.66)	0.43
Hypertension (ref: no)	-0.69 (-1.39, 0.02)	0.06	**-1.05 (-1.75, -0.34)**	**0.004**
Hyperlipidemia (ref: no)	**-1.22 (-1.76, -0.67)**	**<0.0001**	**-0.92 (-1.39, -0.46)**	**0.0001**
Renal failure (ref: no)	**0.71 (0.01, 1.42)**	**0.048**	0.58 (-0.15, 1.31)	0.12
Heart failure (ref: no)	**2.10 (0.42, 3.79)**	**0.01**	**2.03 (0.07, 3.98)**	**0.04**
Gout (ref: no)	**-0.85 (-1.64, -0.05)**	**0.04**	**-0.86 (-1.65, -0.07)**	**0.03**
Diabetes (ref: no)	**0.80 (0.14, 1.45)**	**0.02**	**0.87 (0.20, 1.54)**	**0.01**
COPD (ref: no)	-0.15 (-0.87, 0.56)	0.67	-0.35 (-1.07, 0.38)	0.34
Osteoarthritis (ref: no)	**-0.93 (-1.56, -0.31)**	**0.003**	-0.56 (-1.18, 0.05)	0.07

CHD, coronary heart disease; COPD, chronic obstructive pulmonary disease;

Significant beta coefficients are in bold.

In multivariable-adjusted analyses, age 50 to <65 years, metropolitan teaching hospital status, hospital location in the West region and diabetes were associated with higher total (hospital plus ED) charges (Table 5). Hyperlipidemia, hypertension, gout and osteoarthritis were associated with lower total charges. Sensitivity analyses that examined the logarithm of duration of hospital stay or total charges (normally distributed rather than slightly skewed) showed results similar to the main analysis, indicating that slight skewness of untransformed variables did not impact results.

**Table 5:** Predictors of the total hospital charges (inpatient +ED charges) among patients with septic arthritis who had a hospital admission with septic arthritis after presenting to ER.

	Univariate		Multivariable-adjusted	
	Beta-estimate (95% CI)	P-value	Beta-estimate (95% CI)	P-value
Age				
<50	Ref		Ref	
50- <65	**8,610 (2,123, 15,097)**	**0.009**	**11,693 (5,031, 18,356)**	**0.0006**
65- <80	2,622 (-4,060, 9,305)	0.44	10,037 (-2,026, 22,100)	0.10
≥80	2,291 (-12,354, 16,937)	0.76	11,034 (-11,025, 33,094)	0.33
Gender				
Female (ref)	Ref		Ref	
Male	-1,952 (-7,876, 3,972)	0.52	-1,956 (-7,327, 3,416)	0.47
Median household income				
1st quartile (< $38,999)	Ref		Ref	
2nd quartile ($39,000 to $47,999)	3,341 (-5,339, 12,021)	0.45	3,875 (-5,084, 12,834)	0.40
3rd quartile ($48,000 to $62,999)	**8,832 (997, 16,667)**	**0.03**	6,770 (-1,077, 14,616)	0.09
4th quartile ($63,000 or more)	2,059 (-5,490, 9,609)	0.59	-1,251 (-9,620, 7,118)	0.77
Primary payer				
Medicare	Ref		Ref	
Medicaid	8,683 (-302, 17,668)	0.06	8,569 (-739, 17,877)	0.07
Private insurance	-4,915 (-10,526, 696)	0.09	-3,606 (-10,127, 2,915)	0.28
Self-pay/No charge	2,331 (-7,842, 12,504)	0.65	3,440 (-7,008, 13,887)	0.52
Other	15,966 (-3,159, 35,092)	0.10	9,067 (-9,163, 27,298)	0.33
Patient residence				
Micropolitan/not metro	Ref		Ref	
Metropolitan (large or small)	**17,648 (11,580, 23716)**	**<0.0001**	**12,149 (6,074, 18,225)**	**0.0001**
Hospital Region				
Northeast	Ref		Ref	
Midwest	**-10,239 (-19,766, -712)**	**0.03**	-9,367 (-19,229, 494)	0.06
South	-2,217 (-12,928, 8,493)	0.68	-1,362 (-12,950, 10,227)	0.82
West	**19,194 (5,397, 32,990)**	**0.006**	**18,889 (5,331, 32,448)**	**0.006**
Teaching status of hospital				
Metropolitan non -teaching or non-metro	Ref		Ref	
Metropolitan teaching	6,549 (-1,731, 14,829)	0.12	6,159 (-2,720, 15,039)	0.17
Comorbidities				
CHD (ref: no)	1,525 (-5,950, 8,999)	0.69	1,928 (-6,970, 10,826)	0.67
Hypertension (ref: no)	-4,774 (-10,706, 1,158)	0.11	**-8,889 (-17,153, -624)**	**0.03**
Hyperlipidemia (ref: no)	**-7,526 (-12,530, -2,522)**	**0.003**	**-6,005 (-11,014, -997)**	**0.02**
Renal failure (ref: no)	6,421 (-225, 13,067)	0.06	5,094 (-1,804, 11,992)	0.15
Heart failure (ref: no)	**13,981 (2,351, 25,612)**	**0.02**	12,542 (-1,141, 26,225)	0.07
Gout (ref: no)	**-10,083 (-15,442, -4,723)**	**0.0002**	**-11,127 (-17,105, -5,150)**	**0.0003**
Diabetes (ref: no)	**8,981 (2,532, 15,430)**	**0.006**	**9,144 (3822, 14,466)**	**0.0008**
COPD (ref: no)	-5,122 (-11,623, 1,380)	0.12	-5,127 (-12,568, 2,313)	0.18
Osteoarthritis (ref: no)	**-8,356 (-13,404, -3,308)**	**0.001**	**-7,289 (-14,164, -413)**	**0.04**

CHD, coronary heart disease; COPD, chronic obstructive pulmonary disease

Significant beta coefficients are in bold

### Predictors of discharge to home after hospital admission for septic arthritis

In multivariable-adjusted analyses, gender was no longer statistically significantly associated with the risk of discharge to home. Age >50 years, higher annual household income of $48K-$62.9K (compared with <38K), heart failure, diabetes and a longer hospital stay were associated with lower odds of discharge to home after an ED visit for septic arthritis (Table 6). Private insurance, self-pay or other primary payer (compared to Medicare), Western U.S. location, and gout were associated with higher odds of discharge to home from the hospital (Table 6).

**Table 6:** Predictors of discharge to home among patients who had a hospital admission with septic arthritis after presenting to ER.

	Univariate		Multivariable-adjusted	
	Odds Ratio (95% CI)	P-value	Odds Ratio (95% CI)	P-value
Age				
<50	Ref			
50- <65	**0.46 (0.38, 0.55)**	**<0.0001**	**0.58 (0.47, 0.72)**	**<0.0001**
65- <80	**0.30 (0.25, 0.37)**	**<0.0001**	**0.55 (0.41, 0.75)**	**0.0001**
≥80	**0.18 (0.14, 0.24)**	**<0.0001**	**0.33 (0.23, 0.47)**	**<0.0001**
Gender				
Female	Ref		Ref	
Male	**1.43 (1.23, 1.66)**	**<0.0001**	1.13 (0.96, 1.34)	0.14
Median household income				
1st quartile (< $38,999)	Ref		Ref	
2nd quartile ($39,000 to $47,999)	1.04 (0.85, 1.27)	0.68	1.03 (0.82, 1.29)	0.78
3rd quartile ($48,000 to $62,999)	**0.76 (0.61, 0.94)**	**0.01**	**0.79 (0.62, 1.00)**	**0.045**
4th quartile ($63,000 or more)	0.90 (0.71, 1.14)	0.37	0.99 (0.75, 1.30)	0.94
Primary payer				
Medicare	Ref		Ref	
Medicaid	**1.74 (1.40, 2.17)**	**<0.0001**	1.14 (0.86, 1.51)	0.37
Private insurance	**2.28 (1.89, 2.75)**	**<0.0001**	**1.44 (1.11, 1.86)**	**0.007**
Self-pay/No charge	**6.12 (4.49, 8.33)**	**<0.0001**	**3.44 (2.34, 5.04)**	**<0.0001**
Other	**4.26 (3.03, 5.99)**	**<0.0001**	**2.61 (1.77, 3.84)**	**<0.0001**
Patient location (residence)				
Micropolitan/not metro	Ref		Ref	
Metropolitan (large or small)	0.88 (0.70, 1.10)	0.26	0.83 (0.64, 1.08)	0.16
Hospital Region				
Northeast	Ref		Ref	
Midwest	1.19 (0.92, 1.54)	0.18	1.11 (0.83, 1.48)	0.48
South	**1.42 (1.11, 1.80)**	**0.005**	1.14 (0.86, 1.51)	0.37
West	**1.68 (1.28, 2.22)**	**0.0002**	**1.40 (1.04, 1.89)**	**0.03**
Teaching status of hospital				
Metropolitan non -teaching or non-metro	Ref		Ref	
Metropolitan teaching	1.02 (0.86, 1.22)	0.79	1.05 (0.87, 1.26)	0.63
Comorbidities				
CHD (ref: no)	**0.49 (0.39, 0.61)**	**<0.0001**	0.91 (0.70, 1.18)	0.47
Hypertension (ref: no)	**0.63 (0.55, 0.72)**	**<0.0001**	1.13 (0.96, 1.33)	0.14
Hyperlipidemia (ref: no)	**0.67 (0.57, 0.79)**	**<0.0001**	1.00 (0.82, 1.21)	0.99
Renal failure (ref: no)	**0.56 (0.46, 0.69)**	**<0.0001**	1.00 (0.78, 1.27)	0.97
Heart failure (ref: no)	**0.37 (0.29, 0.49)**	**<0.0001**	**0.71 (0.54, 0.95)**	**0.02**
Gout (ref: no)	1.01 (0.79, 1.30)	0.92	**1.31 (1.02, 1.70)**	**0.04**
Diabetes (ref: no)	**0.55 (0.47, 0.65)**	**<0.0001**	**0.70 (0.58, 0.85)**	**0.0003**
COPD (ref: no)	**0.52 (0.39, 0.68)**	**<0.0001**	0.80 (0.59, 1.09)	0.15
Osteoarthritis (ref: no)	**0.56 (0.45, 0.69)**	**<0.0001**	0.83 (0.65, 1.06)	0.14
Length of stay, in days	**0.96 (0.94, 0.98)**	**0.0005**	**0.96 (0.93, 0.98)**	**0.0002**

CHD, coronary heart disease; COPD, chronic obstructive pulmonary disease

Significant odds ratios are in bold.

### Discussion

We performed a cohort study using a national sample of Americans who were hospitalized with a primary diagnosis of septic arthritis. To our knowledge, our study is the first to provide U.S. national estimates for septic arthritis hospitalizations, associated burden and outcomes. These results are a resource for policy makers, clinicians and researchers. Findings from our study form a basis for further research and investigations into septic arthritis, an important clinical problem, associated with significant morbidity and mortality.

We examined time-trends in hospital length of stay and total hospital charges. We found that total charges for septic arthritis increased by 26% in 2012 compared to 2009; inpatient charges increased 24% from 2009 to 2012. There was a slight increase in the absolute number of hospitalizations due to septic arthritis from 13,087 in 2009 to 13,714 in 2012, but the relative utilization compared to all hospitalizations (over 4 million in 2009 vs. over 4.5 million in 2012) remained stable at 0.32% in 2009 vs. 0.31% in 2012 [4, 5]. We did not observe any time-trends in disposition from hospital to home vs. other settings over time. Compared to patients with ED visits with septic arthritis who were not hospitalized, those with ED visits that were hospitalized were likely to be older, female, metropolitan residents, in higher income quartile, have Medicare as payer, seen at teaching hospital and to have any of the nine comorbidities. We made several other novel observations that merit further discussion.

To our knowledge, this is among the first studies to describe factors associated with longer hospital stay for septic arthritis using a U.S. national sample. Age >50 years, Medicaid and self-pay as primary payer (compared to Medicare), hospital location in the Northeast region, a teaching hospital status, heart failure and diabetes were associated with a longer hospital stay. On the other hand, hyperlipidemia, hypertension or gout were each associated with a shorter hospital stay. Similarly, age 50 to <65 years (compared to <50 years), metropolitan residency, hospital location in West U.S. and diabetes were associated with higher total hospital charges, where as hyperlipidemia, hypertension, osteoarthritis or gout were each associated with a lower hospital charges. It is not surprising that older age and diabetes were associated with both higher hospital charges and longer hospitalization stay. Both factors are associated with immune system modulation that makes an individual not only more susceptible to septic arthritis, but also more likely to have poorer outcome after septic arthritis [7, 8]. We believe that our study is first to identify heart failure as a predictor of longer hospital stay for patients with septic arthritis. We are not sure of the underlying mechanism of this association, but increased fluid load with antibiotic and other intravenous fluid infusions may exacerbate heart failure prolonging hospitalization. Whether heart failure can impact the severity of or recovery from septic arthritis is not well-known.

An interesting study finding was that the presence of gout or osteoarthritis in patients with septic arthritis was associated with a shorter stay or lower hospital charges. While either are known as risk factors for septic arthritis, the presence of a known diagnosis of gout or osteoarthritis, may trigger a higher degree of suspicion for a diagnosis of septic arthritis, and a higher likelihood of earlier arthrocentesis and/or rheumatology consultation. This can in turn help in an earlier identification of the causative organism, leading to an earlier institution of the appropriate antibiotic use rather than broad coverage, thus shortening the hospital stay and reducing charges.

We noted a longer hospital stay in patients with Medicaid or self pay as primary payer or those receiving care at a teaching hospital. One can conceive that more complicated septic arthritis patients may seek care at tertiary care centers or be referred to them, explaining worse outcomes. Patients receiving Medicaid may have lower socio-economic status than comparators and other comorbid illnesses that can negatively impact their outcomes.

Patients with gout were more likely to be discharged home, as were those with Medicare as the primary payer. It is possible the presence of gout allows an earlier diagnosis and treatment allowing discharge to home; or presence of gout indicates a better phenotype that allows patients to recover faster from the treatments of septic arthritis. A longer hospital stay was associated with lower likelihood of discharge to home. This finding is somewhat intuitive since a longer hospital stay might indicate a difficult to treat infection and/or a weak host. Our finding of poorer outcome is similar to one recently noted for hip fracture [9].

Our findings must be interpreted considering study limitations. NEDS collects data at event-level, rather than at patient-level, and does not allow prospective follow-up. Therefore, patients can have multiple visits in a given year. Misclassification bias is possible, since we used an ICD-9-CM code for septic arthritis, which we anticipate would bias findings towards null. However, this code has been shown to be valid in another study [6]. Another limitation is that the diagnosis of septic arthritis may not have been confirmed by a positive gram stain or positive culture in conjunction with clinical features, when the patient is admitted from the ED. In addition, some people may have been admitted due to high clinical suspicion of septic arthritis, but actually may have had a gout flare or musculoskeletal injury or bursitis/tendinitis masquerading as septic arthritis. Our study examined hospital charges, not true costs, which are expected to be lower, since charges are often inflated. NEDS provides data on charges, not actual cost. Lack of laboratory test results (blood or joint fluid gram stain or cultures) or clinical notes in the NEDS data limited us from doing interesting analyses for reasons for higher vs. lower charges and shorter vs. longer stay; these need to be explored in future studies, where these data are available. A test of significance for time-trends in utilization, charges etc. could not be performed due to availability of limited date points. Diagnostic misclassification for secondary diagnoses listed (gout etc.) is also possible, since these were also based on ICD-9-CM codes. Our NEDS database lacked data on disease severity, disease stage and current medications for comorbidities; future studies should assess these variables and their effect on outcomes of septic arthritis.

Our study has several strengths. We used the NEDS data, which is the largest and a truly representative national database of all U.S. visits to ED and subsequent hospitalizations. NEDS also provided us with important patient and hospital covariates and confounders, which were adjusted in the multivariable-adjusted analyses we performed, thereby reducing the possibility of residual confounding.

### Conclusions

In conclusion, in a national, representative U.S. sample of hospitalizations, we studied the time-trends in incidence, duration and total charges for hospitalizations for septic arthritis. We found no significant time-trends, except increasing charges. We described important demographic, comorbidity and hospital characteristic correlates of a longer hospital stay and higher total charges for patients with a primary diagnosis of septic arthritis, in a national sample. Our study found patient age, comorbidity and hospital location were associated with odds of discharge to home among patients hospitalized with septic arthritis. Future studies should examine if comorbidity management or systems interventions can reduce the septic arthritis associated utilization or outcomes.

## Abbreviations

AHRQ: Agency for Healthcare Research and Quality
CHD: coronary heart disease
COPD: chronic obstructive pulmonary disease
ED: Emergency Department
HCUP: Healthcare Cost and Utilization Project
ICD-9-CM: International Classification of Diseases, Ninth revision, Common Modification
NEDS: National Emergency Department Sample
OA: osteoarthritis
